# The economic impact of two diagnostic strategies in the management of restorations in primary teeth: a health economic analysis plan for a trial-based economic evaluation

**DOI:** 10.1186/s13063-021-05722-7

**Published:** 2021-11-12

**Authors:** Raíza Dias Freitas, Bruna Lorena Pereira Moro, Laura Regina Antunes Pontes, Haline Cunha Medeiros Maia, Ana Laura Passaro, Rodolfo Carvalho Oliveira, Jonathan Rafael Garbim, Maria Eduarda Franco Vigano, Tamara Kerber Tedesco, Christopher Deery, Daniela Prócida Raggio, Maximiliano Sergio Cenci, Fausto Medeiros Mendes, Mariana Minatel Braga, CARDEC collaborative group - CARDEC-03 trial, Ana Laura Passaro, Annelry Costa Serra, Antonio Carlos Lopes Silva, Bruna Lorena Pereira Moro, Carolina de Picoli Acosta, Caroline Mariano Laux, Cíntia Saori Saihara, Daniela Prócida Raggio, Fausto Medeiros Mendes, Haline Cunha Medeiros Maia, Isabel Cristina Olegário da Costa, Isabella Ronqui de Almeida, Jhandira Daibelis Yampa Vargas, Jonathan Rafael Garbim, José Carlos P. Imparato, Julia Gomes Freitas, Karina Haibara De Natal, Laura Regina Antunes Pontes, Mariana Bifulco, Mariana Minatel Braga, Mariana Pinheiro de Araújo, Mayume Amorim do Vale, Raiza Dias Freitas, Renata Marques Samuel, Rita Baronti, Rodolfo de Carvalho Oliveira, Simone Cesar, Tatiane Fernandes Novaes, Tamara Kerber Tedesco, Thais Gimenez, Tathiane Larissa Lenzi, Cacia Signori, Maximiliano Sérgio Cenci, Kim Rud Ekstrand

**Affiliations:** 1 Department of Pediatric Dentistry, School of Dentistry, University of São Paulo, Lineu Prestes Avenue, 2227, São Paulo, SP 05508000 Brazil; 2 School of Clinical Dentistry, University of Sheffield, Sheffield, UK; 3 Graduate Program in Dentistry, Federal University of Pelotas, Pelotas, Rio Grande do Sul Brazil

**Keywords:** Cost-effectiveness analysis, Cost-utility analysis, Dental caries

## Abstract

**Background:**

Different approaches have been used by dentists to base their decision. Among them, there are the aesthetical issues that may lead to more interventionist approaches. Indeed, using a more interventionist strategy (the World Dental Federation - FDI), more replacements tend to be indicated than using a minimally invasive one (based on the Caries Around Restorations and Sealants—CARS). Since the resources related to the long-term health effects of these strategies have not been explored, the economic impact of using the less-invasive strategy is still uncertain. Thus, this health economic analysis plan aims to describe methodologic approaches for conducting a trial-based economic evaluation that aims to assess whether a minimally invasive strategy is more efficient in allocating resources than the conventional strategy for managing restorations in primary teeth and extrapolating these findings to a longer time horizon.

**Methods:**

A trial-based economic evaluation will be conducted, including three cost-effectiveness analyses (CEA) and one cost-utility analysis (CUA). These analyses will be based on the main trial (CARDEC-03/NCT03520309), in which children aged 3 to 10 were included and randomized to one of the diagnostic strategies (based on FDI or CARS). An examiner will assess children’s restorations using the randomized strategy, and treatment will be recommended according to the same criteria. The time horizon for this study is 2 years, and we will adopt the societal perspective. The average costs per child for 24 months will be calculated. Three different cost-effectiveness analyses (CEA) will be performed. For CEAs, the effects will be the number of operative interventions (primary CEA analysis), the time to these new interventions, the percentage of patients who did not need new interventions in the follow-up, and changes in children’s oral health-related quality of life (secondary analyses). For CUA, the effect will be tooth-related quality-adjusted life years (QALYs). Intention-to-treat analyses will be conducted. Finally, we will assess the difference when using the minimally invasive strategy for each health effect (∆effect) compared to the conventional strategy (based on FDI) as the reference strategy. The same will be calculated for related costs (∆cost). The discount rate of 5% will be applied for costs and effects. We will perform deterministic and probabilistic sensitivity analyses to handle uncertainties. The net benefit will be calculated, and acceptability curves plotted using different willingness-to-pay thresholds. Using Markov models, a longer-term economic evaluation will be carried out with trial results extrapolated over a primary tooth lifetime horizon.

**Discussion:**

The main trial is ongoing, and data collection is still not finished. Therefore, economic evaluation has not commenced. We hypothesize that conventional strategy will be associated with more need for replacements of restorations in primary molars. These replacements may lead to more reinterventions, leading to higher costs after 2 years. The health effects will be a crucial aspect to take into account when deciding whether the minimally invasive strategy will be more efficient in allocating resources than the conventional strategy when considering the management of restorations in primary teeth. Finally, patients/parents preferences and consequent utility values may also influence this final conclusion about the economic aspects of implementing the minimally invasive approach for managing restorations in clinical practice. Therefore, these trial-based economic evaluations may bring actual evidence of the economic impact of such interventions.

**Trial registration:**

NCT03520309. Registered May 9, 2018. Economic evaluations (the focus of this plan) are not initiated at the moment.

**Supplementary Information:**

The online version contains supplementary material available at 10.1186/s13063-021-05722-7.

## Background

Reinterventions in restored teeth are common procedures in dentistry. Caries lesions around restorations, frequently denominated as “secondary caries,” have been identified as the main reason for repairing or replacing the restorations in primary teeth [1]. The detection of these lesions and other aspects related to defective restorations in primary teeth is challenging as it involves a clinical inspection of the dental surface and the restorative material as well as their interface. The visual-tactile method is commonly used for this purpose. Some clinical strategies based on this method have been proposed to standardize the clinical assessment of restorations and support treatment decisions [2].

In general, dentists base their decision on different parameters, including aesthetical ones. This option tends to result in a more interventionist approach. In 2007, the World Dental Federation (FDI) proposed a strategy to evaluate restorations comprising aesthetic, functional, and biological parameters, including the presence of caries and related aspects [3]. The FDI criteria were proposed for research and clinical practice and used to decide reintervention in restored teeth [4]. Due to the several aesthetic parameters evaluated, the diagnostic strategy based on FDI embraces a cosmetic dentistry perspective, relating to a more interventionist approach for the clinical practice.

On the other hand, the caries associated with restorations and sealants (CARS) strategy is a more recently minimally invasive strategy proposed as part of the International Caries Classification and Management System (ICCMS) [5] and exclusively focused on detecting caries lesions around the restorations [6]. The CARS strategy is based on the International Caries Detection and Assessment System (ICDAS) scores. It is more consistent with a Cariology background, leaning on a less interventionist approach, based solely on the occurrence of caries lesions and their characteristics.

To date, there is no consensus on the best strategy to adopt in clinical practice, and most studies do not explore the clinical relevance of the accuracy tests nor patient-centered outcomes [2]. An ongoing clinical trial (CARies DEtection in Children - CARDEC-03) aims to assess the impact of using the FDI and CARS criteria in the assessment of restorations in primary teeth [7]. At first glance, when using a more interventionist strategy (using the FDI criteria), the indication of replacements of restorations in the baseline was more frequent than using the strategy based on CARS [8]. Nevertheless, the resources related to the long-term health effects have not been explored yet.

When defective restorations in primary teeth need to be assessed to guide their management, it is not known if this minimally invasive strategy is efficient for allocating resources compared to the conventional strategy, based on FDI criteria. Even if the diagnostic method benefits patients, the subsequent financial impact should be assessed, featuring phase 5 studies for diagnostic methods [9]. As dental expenditure was $298 billion in 2010, representing 4.6% of global healthcare costs [10], economic evaluations to direct resources to the best diagnostic strategies are critical for clinical practice to be financially viable. On the other hand, economic evaluations assessing diagnostic strategies are scarce and, in several cases, are not standardized and present low quality [11].

We are presenting a health economic analysis plan to guide a trial-based economic evaluation. The publication of the health economic analysis plan has been becoming the best practice for trial-based economic assessments. Publishing an economic analysis plan is currently relevant since it increases the reproducibility, dissemination to other research groups, and transparency of the analyses. Indeed, this process intends to guarantee that the process avoids selection bias related to data sources and valuation methods, selective reporting in results, and the use of unplanned analyses to satisfy a specific hypothesis [12, 13]. The present health economic analysis plan aims (1) to describe methodologic strategies for conducting a transparent trial-based economic evaluation that aims to assess whether a minimally invasive strategy is more efficient in allocating resources than the conventional strategy for managing restorations in primary teeth and (2) to construct a decision analytic modelling framework to extrapolate these findings considering a primary molar lifetime horizon.

## Methods

This manuscript is a health economic analysis plan following the International Society for Pharmacoeconomics and Outcomes Research (ISPOR) Good Research Practices Task Force Report recommendations [14] and the Consolidated Health Economic Evaluation Reporting Standards (CHEERS) [15] checklist.

### Study design

A trial-based economic evaluation will be conducted (piggyback approach), including three cost-effectiveness analyses (CEA)—different health effects—and a cost-utility analysis (CUA). The clinical trial investigating the diagnostic strategy for restorations assessment is the third diagnostic study conducted by the CARDEC collaborative group at the School of Dentistry of the University of São Paulo (São Paulo, Brazil). The CARDEC-03 trial is a two-arm, parallel-group, patient-randomized controlled trial aiming to assess which diagnostic strategies (based on FDI criteria or CARS) leads to fewer new interventions in restored primary teeth during 2 years of follow-up. Further details regarding the trial have been published in the study protocol [7].

The strategy based on the FDI criteria will be acknowledged as the reference strategy for assessing the restorations. However, recognize there is no robust evidence supporting this assumption. Despite this, a reference strategy for economic evaluation must be assumed. Considering that the CARS strategy is associated with a less interventionist approach, we will consider it as the new strategy. Moreover, FDI criteria were first proposed and appointed by experts as the standard criteria for restorations’ assessment [16, 17].

### Target population and eligibility criteria

Children’s participation was voluntary. Our sample includes 3-to-10-year-old children seeking dental care at the Pediatric Dentistry clinic from our school, with at least one dental restoration in a primary tooth. The exclusion criteria were children whose parents did not consent to their participation, children who did not assent participating in this study, and children with limited ability to co-operate even when behavior management was used [18].

### Comparators—interventions and follow-up

Aiming to compare a more interventionist strategy to a supposedly less interventionist approach when assessing dental restorations and guiding clinical decision-making, children were allocated to one of the two diagnostic strategies for the assessment of restorations. To simplify, we will refer to them, from that point, as FDI and CARS strategies. A trained and calibrated examiner (BLPM) performed the assessments, and treatment decisions were based on the criteria. The FDI criteria [16] can be adapted depending on the purpose of the study. Therefore, since dental caries is the most common reported reason for reinterventions in primary teeth, we chose to evaluate related parameters as marginal staining and adaptation, besides the recurrence of caries. The CARS strategy will be used as originally proposed [5] (Table 1). Details regarding clinical criteria, sample size, randomization, allocation, blinding, and treatment of the restorations have been previously described in a clinical trial protocol [7].

**Table 1:**
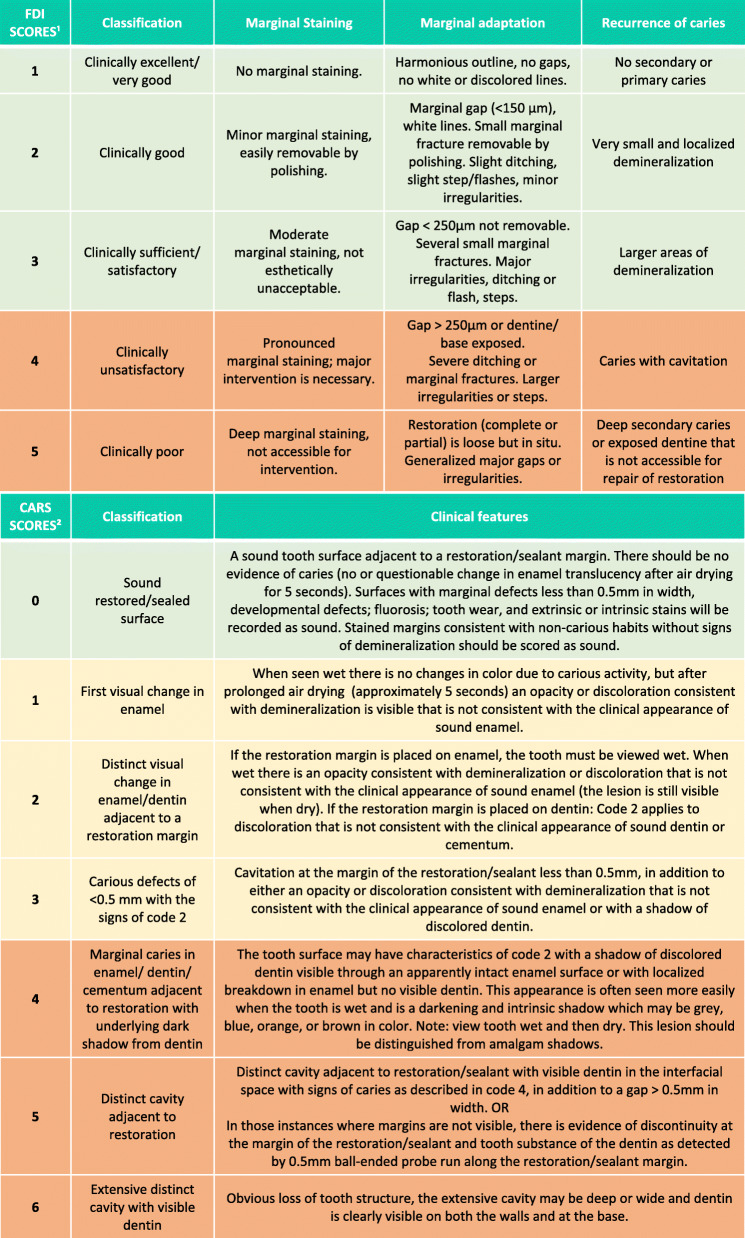
Clinical strategies for FDI and CARS criteria—adapted from Moro et al. [7]

^1^Based on Hickel et al. [16]

^2^Based on Pitts et al. [14]

Children will be followed for 24 months after the baseline interventions. Clinical assessments are being scheduled at 6-month intervals. In the baseline and at each follow-up visit, children are being instructed about diet and oral hygiene. The same examiner responsible for baseline evaluation will reassess the restorations at each appointment and propose a new treatment plan for each child based on the randomized strategy.

### Time horizon, study perspective, and discount rate

The time horizon for the main evaluations was set as 24 months (time of study enrollment). Secondary longer-term economic evaluation with trial results will be performed to extrapolate the results over a primary tooth lifetime horizon. We will adopt the societal perspective, accounting for direct and indirect costs. A discount rate of 5% will be applied for costs and effects as the trial is being conducted in Brazil, a lower-middle-income country [19]. Further sensitivity analyses will test the influence of this assumption by considering different discount rates (0–10%).

### Costs and resources

The costs of each strategy will be estimated using a micro-costing approach. The direct and indirect costs per tooth and child will be calculated over 24 months (Supplemental Material 1). Direct costs will comprise the expenses related to the dental office accommodation, dental instruments and equipment and their respective maintenance, materials used to implement the strategies, and staff expenses (based on working hours and time spent on patient’s care). Firstly, direct costs will be estimated per tooth included in the trial. Then, we will sum up all the child’s eligible teeth for calculating direct costs with each child.

We will calculate the accommodation costs using rental costs and municipal taxes per square meter of the area used by each dental unit. Subsequently, the accommodation costs per hour will be calculated. The same calculation will be used for dental instruments and equipment, estimating a life span of 3 years for instruments [20] and 5 years for equipment [21], with a monthly usage of 160 h. The staff salary (dentists and dental auxiliaries) will be calculated based on the Brazilian Federal Law’s monthly wage, allowing 40 h per week (8 h/day) for each dentist and dental nurse. For dental material, we will calculate the mean value of each item in three different dental stores and quantities used during clinical appointments.

Indirect costs will include out-of-pocket expenditures, such as transportation (public or private), any opportunity costs of accompanying a person’s absence from the workplace, and the patient’s time accessing care. These costs will be estimated per child, considering the time spent during appointments and waiting or travelling to/from the dental clinic. For indirect costs per tooth, time spent performing procedures related to each specific tooth will be first considered. For the child’s general appointments (e.g., instructions, fluoride applications) and the child’s and accompanying person’s waiting/travelling, the time spent will be fully considered for each tooth, as if only one tooth had been included per child. Possible dental interventions received externally to the research, but related to the assessed teeth, will also be considered indirect costs.

Transportation costs will be calculated using the municipality’s fares for public transportation. For private transport, we will consider the distance from the family’s house to the University and an average price for fuel obtained from the Brazilian National Agency, assuming an 8 km per liter efficiency. The patient’s and accompanying person’s time will be valued, respectively, based on the Brazilian minimum wage and mean Brazilian salary. Suppose the accompanying person reports any earning loss due to being present at the child’s appointments, an additional cost of a working day will be added for each appointment the child attends. The accompanying person’s working absence time will also be calculated based on the mean Brazilian salary. In this case, the working days and hours will be considered to estimate this person’s value per working hour.

To estimate the costs, we have registered in a specific form the number of appointments, the time spent at each one, and materials used during patient care (Supplemental Material 2). This form has also been used to collect information about transportation and absence from work. Details about the cost estimation of each of the resources mentioned above can be found in Supplemental Material 1.

Costs will be calculated in Brazilian Real (BRL) considering the base year for the analysis and converted to international dollars using purchasing power parities (PPP) measured for the same period (or the most recent indicator available at the time of the analyses).

### Health outcomes

Three health effects will be considered for different CEAs to bring different perspectives when decision-making. The primary health effect considered will be the number of new operative interventions per child after the baseline assessment. Other endpoints were set as secondary health effects: the time to the new operative interventions (survival), the percentage of children who did not need new operative interventions, and the relevant change in the Oral Health-Related Quality of Life (OHRQoL) scores (Table 2).

**Table 2:**
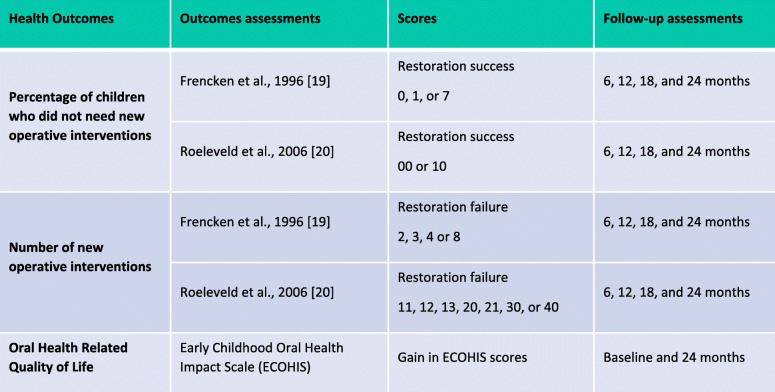
Summary of health outcomes (health effects) used in economic evaluations

95%CI* bootstrap-adjusted confidence interval at 95%

For the first health outcomes (related to new operative interventions), we will assess the children for 24 months, following them each 6 months. The cumulative result will be accumulated for 24 months when computing the number of events (new interventions) and the time to an event during this period. The restorations will be evaluated by an examiner (TKT), blinded to the diagnostic strategy. At this assessment, surfaces were scored according to the restoration integrity and occurrence of caries, determining the need (or not) of repair, replacement, or other possible new interventions [22, 23] (Table 2). At this stage, the idea was to use an external assessor using a different approach (from those interventions under comparison and randomized) not to bias the outcome assessment. Based on this assessment, new interventions (events) will be considered when any need for restoration repair or replacement is identified, any presence of secondary caries lesion exposing dentin is detected, any need for extension of the existing restoration or endodontic treatment is required (due to caries or tooth fracture), and/or any episode of pain is reported (Table 2).

The OHRQoL will be assessed using the Brazilian version of the Early Childhood Health Impact Scale (B-ECOHIS) [24]. This questionnaire is answered by parents as a proxy of the child’s OHRQoL and is a valid measure for children [25]. Although the ECOHIS has been proposed for pre-school children [26], it was chosen to measure effectiveness in the entire sample, comprising children from 3 to 9 years old. The questionnaire was answered in the baseline and will be answered at 24-month follow-up completion. The difference between the ECOHIS final and baseline scores will be calculated. The change in ECOHIS scores will be classified according to the minimal important difference calculated [25].

For CUA, the effect will be the gain in tooth-related quality-adjusted life years (QALYs). To estimate tooth-related QALYs, we will use the Standard Gamble (SG) approach to calculate weights (utility scores) based on the patient’s parent’s preferences regarding health states related to dental caries. For that, we anchored the weighs in tooth loss (the worst scenario). The parent preference will be used as a proxy measure for the child’s preference regarding different health statuses. More details about the Standard Gamble experiment may be found in the next section.

### Standard gamble

We will conduct an SG experiment to measure different oral health states’ preferences related to dental caries in primary teeth. As parents’ answers will be considered a proxy measurement, a representative sample of those parents seeking dental treatment in a reference center will be selected. A minimum sample size of 50 parents was calculated to permit an absolute difference of 0.05 units and guarantee the power of 80% and a significance level of 5%. To compensate for possible non-normal distribution and possible non-response or lost participants, we added up, respectively, 10% and 20% to this calculated sample, totalizing 63 participants to be recruited.

The recruited sample will be stratified by the child’s caries experience and opportunity for dental treatment (children firstly seeking the treatment vs those already enrolled in treatment) to contribute to the sample representativeness. Part of this sample will be selected among children’s parents from the main clinical trial (CARDEC-3). The other will be recruited among parents whose children are seeking treatment in the school’s dental clinics. Adults will be asked about their preference between two courses of action resulting in different outcomes regarding their child’s oral condition.

**Fig. 1:**
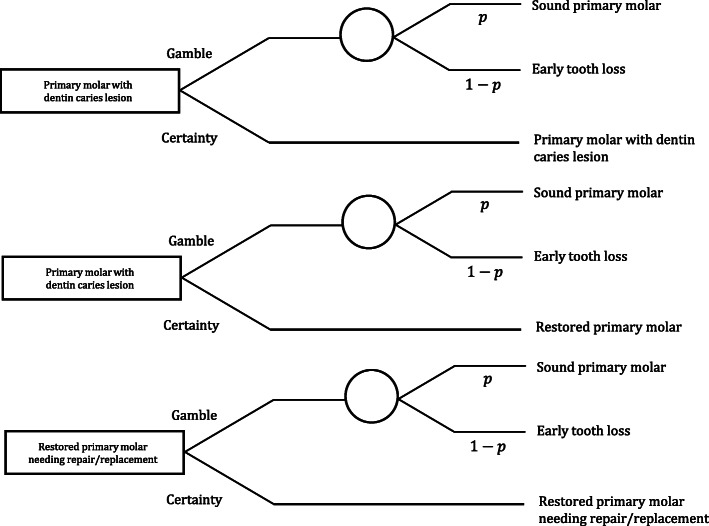
The Standard Gamble experiment to be performed with parents assessing three health states related to dental caries in their child’s primary molars

The health states will be illustrated on cards, and the SG will be conducted using a chance board. The health states considered are (1) a primary molar with dentin caries lesion; (2) a restored primary molar; (3) a restored primary molar needing repair/replacement. Children’s parents will choose between alternatives A and B. Alternative A offers a probability “p” of achieving the best possible health state, which is a sound tooth that will last like that until it exfoliates. Then, a probability “1 − *p*” of having the worst possible condition is assumed (early tooth loss) (Fig. 1). Alternative B will be a particular health state of a restored primary molar. The probability “*p*” will be changed in the chance board until the parent is indifferent to the two options [27]. This probability will be considered the parent’s preference (utility weight) for their child’s health state (utility value). We will then calculate the tooth-related QALYs, also considering the time for which the child presented such a state. The same experiment with the other health states will be conducted, as demonstrated in Fig. 1.

### Analytical methods

The economic evaluations will be considered intention-to-treat analyses using data collected after 2 years, as previously described. In the case of missing data, we will investigate their nature and choose the most appropriate method to handle the missing data, e.g., multiple imputations. Imputations will consider health and economic outcomes and the possible relationship between them and other pertinent covariates. When new operative treatments have been performed externally to the research, the same strategy used for missing data will be used for cost estimation.

Cox regression model with shared frailty will be used to compare the need for a new intervention. The health effects listed above will be compared between groups using the most appropriate statistical test, depending on data distribution. Given the usual right-skewed distribution of cost data, we will use the bootstrapping quantile regression to compare the total costs of the diagnostic strategies [28]. Bootstrapping replications will be set at 1000, and a fixed seed will be determined. We will use the software Stata13 (StataCorp LP, Texas, USA) and set a 5% significance level for these analyses.

We will work with the difference between the strategies both regarding the inputs (∆costs: CARS costs − FDI costs) and outputs (∆effects: CARS effects − FDI effects) since the focus of this series of economic evaluations is the economic impact of using the minimally invasive strategy (based on CARS) instead of the conventional strategy (based on FDI criteria) for managing dental restorations. Bootstrap confidence intervals will be calculated for each parameter considering the costs, effects, incremental costs, and incremental effects [29].

Deterministic one-way sensitivity analysis will be conducted for CEAs and CUA to assess the quantitative relationship among estimates in parameters that could perform differently in a distinct scenario, such as costs, discount rate, and effects. In these analyses, we will also test the influence of different baseline conditions as covariates associated with the effects and costs [30], checking the possibility of extrapolating data from this single trial to a broader population. The results will be demonstrated in a tornado diagram.

Additionally, a Bayesian approach will be used to explore uncertainties on the same parameters. By adopting this approach, we will describe the probabilities around the actual values obtained in this study [31–33]. The data distribution of costs and effects will be checked using XLSTAT Premium 2021.3.1 (Addinsoft, Paris, France), and, based on that distribution, Monte-Carlo simulations (× 10,000) will be generated to be plotted in a cost-effectiveness plane (CE plane). The proportion of points in each quadrant of the CE plane will be calculated, and the location of points will also be assessed visually. We will calculate the incremental net benefit using the following equation:

Incremental Net Benefit = Incremental Effect × Ceiling Ratio − Incremental Cost, being value 1 for a positive coefficient and 0 for a negative coefficient value. Thus, for the interpretation, if the difference is higher than zero (the value 1), it means that for one additional unit of effectiveness, the incremental cost is below the Ceiling Ratio (the maximum value that decision-makers are willing to pay). If the difference is less than zero (the value 0), the incremental cost of each additional unit of effectiveness is above the Ceiling Ratio [34]. Finally, acceptability curves will be plotted for each effect using the incremental net benefit framework and assuming different ceiling ratios to check the uncertainties around threshold points.

Subgroup analyses considering age (3 to 6 vs 7 to 10 years) and patients’ caries experience (≤ 3 vs > 3 restorations) will also be conducted.

### Modelling for primary tooth lifetime horizon

We will construct a decision analytic modelling framework to extrapolate the findings considering a longer time horizon (the primary molar lifetime) (Supplemental File 3). As the base case, we will consider a child as those enrolled in the trial. Then, based on the mean age of children enrolled on the main trial, we will establish the number of cycles of the Markov model.

Probabilities and costs will be extracted from the main trial. If necessary, any additional reference value will be identified from the literature. The SG experiment will generate utility values. We will assume that probabilities will maintain the same at each cycle during the time horizon. The half-cycle correction will be used to account for the fact that events and transitions can occur at any point during the cycle, not necessarily at the start or end of each cycle.

We will adopt the same strategies adopted in the trial-based analyses for deterministic and probabilistic analyses using the model framework. The final interpretation of uncertainties will be considered for this longer time horizon. Data will be modelled and analyzed using a Markov simulation model. Tree Age Pro 2017 (TreeAge Software, Williamstown, MA, USA).

## Discussion

The results from this study will provide necessary evidence regarding the economic impact of the possible implementation of potentially less interventionist diagnostic strategies, such as that based on CARS, when managing restorations in primary teeth. Owing to the lack of high-quality economic evaluation studies in the pediatric dentistry field [11], our study will strengthen the evidence and guide an evidence-informed decision-making process concerning diagnosing dental caries adjacent to restorations in primary teeth. To the best of our knowledge, no study has evaluated the economic impact of diagnostic strategies focused on such a clinical condition.

The strategy based on FDI may lead to a greater number of operative interventions [8], probably due to merge the assessment of the presence of recurrent caries and the restoration staining and adaptation. At first glance, the need for more interventions in the first treatment plan may lead to additional costs since the baseline. However, in a complete economic evaluation, not only costs are considered. Health outcomes are also important in determining the cost-effectiveness of a strategy [35]. Assuming a longer time horizon, we can expect as more interventionist; more reinterventions may be needed, as demonstrated in a previous clinical trial from our group [36]. Then, much higher expenses could have resulted. On the other hand, eventually, depending on how the non-intervened restorations behave during the follow-up, a different scenario may be observed, impacting on effects or not. Since it is an ongoing trial, the long-term health effects (at 2 years) will be crucial to decide, for assessment and management of restorations in primary teeth, whether a minimally invasive strategy (as that based on CARS) will be more efficient in allocating resources than the conventional one (based on FDI criteria).

CEA is one of the most widely used economic evaluations in healthcare, as the effects are clinical measures [35, 37]. We opted to use different parallel economic evaluations at this protocol to bring different perspectives and additional subsidies to decision-makers. In this sense, we considered the primary health effect as the number of new operative interventions. This outcome represents the effect magnitude explored when comparing the diagnostic strategies in the trial. Although other endpoints (effects) have been set as secondary ones, they may show additional views to decision-makers. They offer perspectives regarding the time to the effect, demand for treatment, and patient-centered opinions that may also be helpful when implementing one or another in the health system.

On the other hand, patients/parents preferences and consequent utility values may also influence the final impression about the economic aspects of implementing the minimally invasive strategy, like CARS, in clinical practice. In this sense, CUA would be a valuable tool since it integrates patient-centered care philosophy and should be used when the quality of life is an important outcome [27]. CUA evaluates the effects on qualitative and quantitative health gains, often measured through QALYs. These are the product of time and utility obtained through the patient’s preferences for different health states [27]. As dental caries in children has a relevant impact on quality of life [38], studies involving the economic implications of caries diagnosis and management would benefit from CUA.

Utilities related to health states related to dental caries in primary teeth have been assessed through pre-scored multi-attribute health status classification systems, such as the CHU-9D, or through the visual analog scale (VAS) [39, 40]. One of the main concerns about pre-scored measures is that they may not identify the impact of oral diseases, such as dental caries [41]. Besides, the scaling methods will not necessarily express participants’ sacrifice is willing to take to achieve the health states, and they are more prone to contextual bias [42]. Conversely, the SG is a choice-based method of obtaining “patients” preferences for health states under uncertainty. Although it is time-consuming, the SG is conceptually based on the expected utility theory [43], and it involves the highest sacrifice the participants are willing to take. Finally, in this SG experiment, we could anchor the utility weights in tooth loss, considering it is our worst scenario planned and called the measure derived from it as tooth-related QALY. Although its questionable interchangeability to general QALYs, tooth-related QALY may be a relevant measure for decision-makers in dentistry, especially considering primary teeth, the type of injuries, and their health consequences in children.

Given the SG experiment inherent complexity, we decided to adopt the parents’ valuation of utility as a proxy measurement from the child’s preferences related to their oral health states. This approach has been widely used in studies of children’s preferences [44]. Although these proxy answers have some limitations, it would be a reasonable and feasible approach to a first attempt in determining utility scores related to dental caries, independently of the child’s age. Due to the broader age range in the base clinical trial, we opted for this approach.

Therefore, the results of these trial-based economic evaluations may bring actual evidence about the economic impact of such implementation and contribute to the decision-making process pertaining to the assessment and management of restorations in children. Analytical strategies adopted (e.g., probabilistic sensitivity analyses (scenario) and modelling for primary molars lifespan) may be alternatives to minimize possible limitations in results extrapolation derived from single-studies economic evaluations [45]. In this sense, they may permit that the results are broadly generalized to children seeking dental treatment, who will demand decision and management of their previously placed restorations.

## Trial status

CARDEC-03 trial recruitment took place from November 2017 to November 2018. Each patient will be followed for 24 months. Due to the COVID-19 pandemic situation, our goal is to complete the follow-up by May 2021.

## Supplementary Information

**Additional file 1:.** Supplemental Material 1. Cost items and valuation methods for direct and indirect costs.

**Additional file 2:.** Supplemental Material 2. Form used for resource measurement and subsequent cost estimation.

**Additional file 3:.** Supplemental Material 3. First draft of a theoretical framework to construct an analytic Markov model for modelling strategies for primary tooth lifetime horizon

**Additional file 4:.** EQUATOR network reporting checklist - Consolidated Health Economic Evaluation Reporting Standards (CHEERS) checklist. Note the items related to Results and Discussion (aspects related to findings) are not addressed since this is a health economic analysis plan.

## Data Availability

Data sharing does not apply to this article as no datasets were generated or analyzed during the current study.

## Abbreviations

FDI: World Dental Federation
CARS: Caries around restorations and sealants
CEA: Cost-effectiveness analysis
ECOHIS: Early Childhood Oral Health Impact Scale
CUA: Cost-utility analysis
QALYs: Quality-adjusted life years
ICER: Incremental cost-effectiveness ratio
ICCMS: International Caries Classification and Management System
ICDAS: International Caries Detection and Assessment System
HEAP: Health Economic Analysis Plan
RCT: Randomized controlled trial
ISPOR: International Society for Pharmacoeconomics and Outcomes Research
CARDEC: Caries detection in children
OHRQoL: Oral health-related quality of life
SG: Standard gamble

## References

[1] Chisini LA, Collares K, Cademartori MG, de Oliveira LJC, Conde MCM, Demarco FF, Corrêa MB (2018). Restorations in primary teeth: a systematic review on survival and reasons for failures. Int J Paediatr Dent..

[2] Signori C, Gimenez T, Mendes FM, NJM O, Cenci MS, Huysmans MCDNJM (2018). Clinical relevance of studies on the visual and radiographic methods for detecting secondary caries lesions – a systematic review. J Dent.

[3] Hickel R, Roulet JF, Bayne S, Heintze SD, Mjör IA, Peters M, Rousson V, Randall R, Schmalz G, Tyas M, Vanherle G (2007). Recommendations for conducting controlled clinical studies of dental restorative materials. Clin Oral Investig..

[4] Marquillier T, Doméjean S, Le Clerc J, Chemla F, Gritsch K, Maurin J-C (2018). The use of FDI criteria in clinical trials on direct dental restorations: a scoping review. J Dent..

[5] Pitts NB, Ismail AI, Martignon S, Ekstrand K, Douglas GV V., Longbottom C. ICCMS^TM^ quick reference guide for practitioners and educators. ICCMS^TM^ Resour. 2014;:1–84.

[6] Pitts N, Ekstrand K (2013). International Caries Detection and Assessment System (ICDAS) and its International Caries Classification and Management System (ICCMS) - methods for staging of the caries process and enabling dentists to manage caries. Community Dent Oral Epidemiol..

[7] Moro BLP, Signori C, Freitas RD, Pontes LRA, Lenzi TL, Tedesco TK (2021). The effect of two clinical criteria in the assessment of caries lesions around restorations in children (CARDEC-03): study protocol for a diagnostic randomized clinical trial. F1000Research.

[8] Moro BLP, Freitas RD, Pontes LRA, Pássaro AL, Lenzi TL, Tedesco TK, Ekstrand KR, Braga MM, Raggio DP, Cenci MS, Mendes FM (2020). Influence of different clinical criteria on the decision to replace restorations in primary teeth. J Dent..

[9] Haynes RB, You JJ, Knottnerus JA, Buntinx F (2009). The architecture of diagnostic research. The evidence base of clinical diagnosis.

[10] Rogers HJ, Rodd HD, Vermaire JH, Stevens K, Knapp R, El Yousfi S (2019). A systematic review of the quality and scope of economic evaluations in child oral health research. BMC Oral Health..

[11] Listl S, Galloway J, Mossey PA, Marcenes W (2015). Global economic impact of dental diseases. J Dent Res..

[12] Thorn JC, Davies CF, Brookes ST, Noble SM, Dritsaki M, Gray E, Hughes DA, Mihaylova B, Petrou S, Ridyard C, Sach T, Wilson ECF, Wordsworth S, Hollingworth W (2021). Content of Health Economics Analysis Plans (HEAPs) for trial-based economic evaluations: expert Delphi consensus survey. Value Health..

[13] Dritsaki M, Gray A, Petrou S, Dutton S, Lamb SE, Thorn JC (2018). Current UK Practices on Health Economics Analysis Plans (HEAPs): are we using heaps of them?. Pharmacoeconomics..

[14] Ramsey SD, Willke RJ, Glick H, Reed SD, Augustovski F, Jonsson B, Briggs A, Sullivan SD (2015). Cost-effectiveness analysis alongside clinical trials II - an ISPOR good research practices task force report. Value Heal..

[15] Husereau D, Drummond M, Petrou S, Carswell C, Moher D, Greenberg D, Augustovski F, Briggs AH, Mauskopf J, Loder E (2013). Consolidated Health Economic Evaluation Reporting Standards (CHEERS) statement. Eur J Heal Econ..

[16] Hickel R, Peschke A, Tyas M, Mjör I, Bayne S, Peters M, Hiller KA, Randall R, Vanherle G, Heintze SD (2010). FDI World Dental Federation: Clinical criteria for the evaluation of direct and indirect restorations-update and clinical examples. Clin Oral Investig..

[17] Hickel R, Peschke A, Tyas M, Mjör I, Bayne S, Peters M, Hiller KA, Randall R, Vanherle G, Heintze SD (2010). FDI World Dental Federation - clinical criteria for the evaluation of direct and indirect restorations. Update and clinical examples. J Adhes Dent..

[18] AAPD (2011). Guideline on behavior guidance for the pediatric dental patient: reference manual. Am Acad Pediatr Dent.

[19] Haacker M, Hallett TB, Atun R (2020). On discount rates for economic evaluations in global health. Health Policy Plan..

[20] da Mata C, Allen PF, Cronin M, O’Mahony D, McKenna G, Woods N (2014). Cost-effectiveness of ART restorations in elderly adults: a randomized clinical trial. Community Dent Oral Epidemiol..

[21] Floriano I, Gimenez T, Reyes A, Matos R, Mattos-Silveira J (2013). Análise de custos de diferentes abordagens para avaliação de lesões de cárie em dentes decíduos. Braz Oral Res..

[22] Frencken JE, Makoni F, Sithole WD (1996). Atraumatic restorative treatment and glass-lonomer sealants in a school oral health programme in Zimbabwe: evaluation after 1 year. Caries Res..

[23] Roeleveld AC, van Amerongen WE, Mandari GJ (2006). Influence of residual caries and cervical gaps on the survival rate of Class II glass ionomer restorations. Eur Arch Paediatr Dent..

[24] Martins-Júnior PA, Ramos-Jorge J, Paiva SM, Marques LS, Ramos-Jorge ML (2012). Validations of the Brazilian version of the Early Childhood Oral Health Impact Scale (ECOHIS). Cad Saude Publica..

[25] Novaes TF, Pontes LRA, Freitas JG, Acosta CP, Andrade KCE, Guedes RS (2017). Responsiveness of the Early Childhood Oral Health Impact Scale (ECOHIS) is related to dental treatment complexity. Health Qual Life Outcomes..

[26] Pahel BT, Rozier RG, Slade GD (2007). Parental perceptions of children’s oral health: the Early Childhood Oral Health Impact Scale (ECOHIS). Health Qual Life Outcomes..

[27] Brazier J, Ratcliffe J, Salomon JA, Tsuchiya A (2017). Measuring and valuing health benefits for economic evaluation.

[28] Desgagné A. The use of the bootstrap statistical method for the pharmacoeconomic cost analysis of skewed data. Pharmacoeconomics. 1998;13 5 PART I:487–497. 10.2165/00019053-199813050-00002 10180748

[29] Barber JA, Thompson SG (2000). Analysis of cost data in randomized trials: an application of the non-parametric bootstrap. Stat Med..

[30] Braga M, Machado T, Rocha E, Floriano I, Raggio D, Mendes F. How to extrapolate trial-based economic evaluations to populations? – proposing sensitivity analyses based on national data. In: Caries Research. 2021.

[31] Briggs AH, Gray AM (1999). Handling uncertainty when performing economic evaluation of healthcare interventions. Health Technol Assess (Rockv)..

[32] Sendi P, Gafni A, Birch S (2002). Opportunity costs and uncertainty in the economic evaluation of health care interventions. Health Econ..

[33] Heitjan DF, Moskowitz AJ, Whang W (1999). Bayesian estimation of cost-effectiveness ratios from clinical trials. Health Econ..

[34] Hounton S, Newlands D (2012). Applying the net-benefit framework for assessing cost-effectiveness of interventions towards universal health coverage. Cost Eff Resour Alloc..

[35] Drummond MF, Sculpher MJ, Claxton K, Stoddart GL, Torrance GW (2015). Methods for the economic evaluation of health care programmes. Fourth Edi.

[36] Pontes L, Novaes T, Lara J, Gimenez T, Moro B, Camargo L (2020). Impact of visual inspection and radiographs for caries detection in children: a 2-year randomized clinical trial. J Am Dent Assoc..

[37] Rudmik L, Drummond M (2013). Health economic evaluation: important principles and methodology. Laryngoscope..

[38] Martins MT, Sardenberg F, Bendo CB, Vale P, Paiva SM, Pordeus IA (2017). Dental caries remains as the main oral condition with the greatest impact on children ’ s quality of life. PLoS One..

[39] Tamošiunas V, Kay E, Craven R (2013). A preliminary study applying decision analysis to the treatment of caries in primary teeth. Stomatologija..

[40] Koh R, Pukallus M, Kularatna S, Gordon LG, Barnett AG, Walsh LJ, Seow WK (2015). Relative cost-effectiveness of home visits and telephone contacts in preventing early childhood caries. Community Dent Oral Epidemiol..

[41] Foster Page LA, Beckett DM, Cameron CM, Thomson WM (2015). Can the Child Health Utility 9D measure be useful in oral health research?. Int J Paediatr Dent..

[42] Bleichrodt H, Johannesson M (1997). An experimental test of a theoretical foundation for rating-scale valuations. Med Decis Mak..

[43] Sampson C, Devlin N, Parkin D (2020). Drop dead: is anchoring at ‘dead’ a theoretical requirement in health state valuation? OHE Res Pap.

[44] Wolstenholme JL, Bargo D, Wang K, Harnden A, Räisänen U, Abel L (2018). Preference-based measures to obtain health state utility values for use in economic evaluations with child-based populations: a review and UK-based focus group assessment of patient and parent choices. Qual Life Res..

[45] Sculpher MJ, Claxton K, Drummond M, McCabe C (2006). Whither trial-based economic evaluation for health care decision making?. Health Econ..

