# Supplementary figures and images for: The economic impact of two diagnostic strategies in the management of restorations in primary teeth: a health economic analysis plan for a trial-based economic evaluation
Source: Trials. 2021 Nov 12;22:794. doi: 10.1186/s13063-021-05722-7 (PMC8586840; doi:10.1186/s13063-021-05722-7)

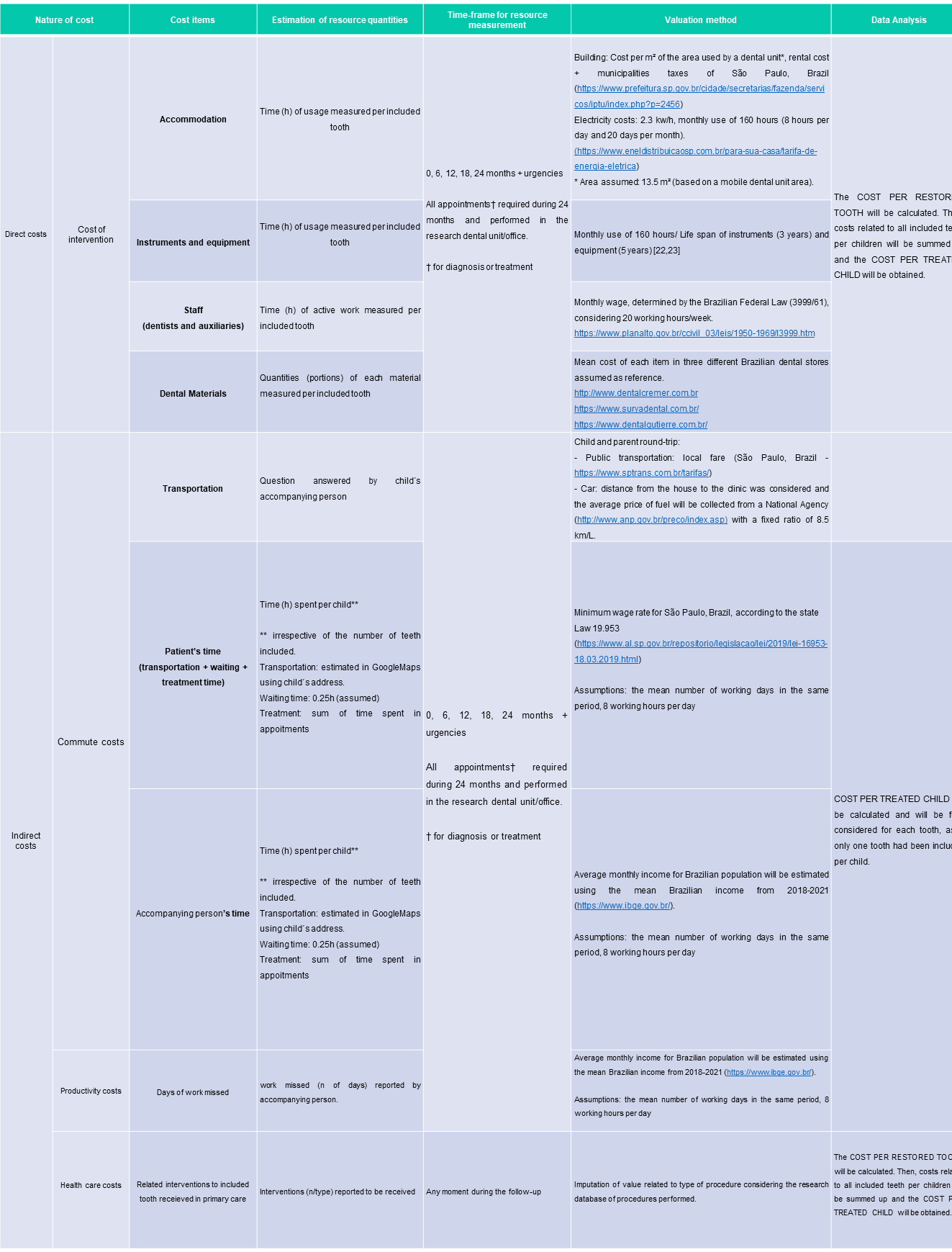

Supplement: Supplementary file 1 — Additional file 1:. Supplemental Material 1. Cost items and valuation methods for direct and indirect costs. [file 13063_2021_5722_MOESM1_ESM.tif]

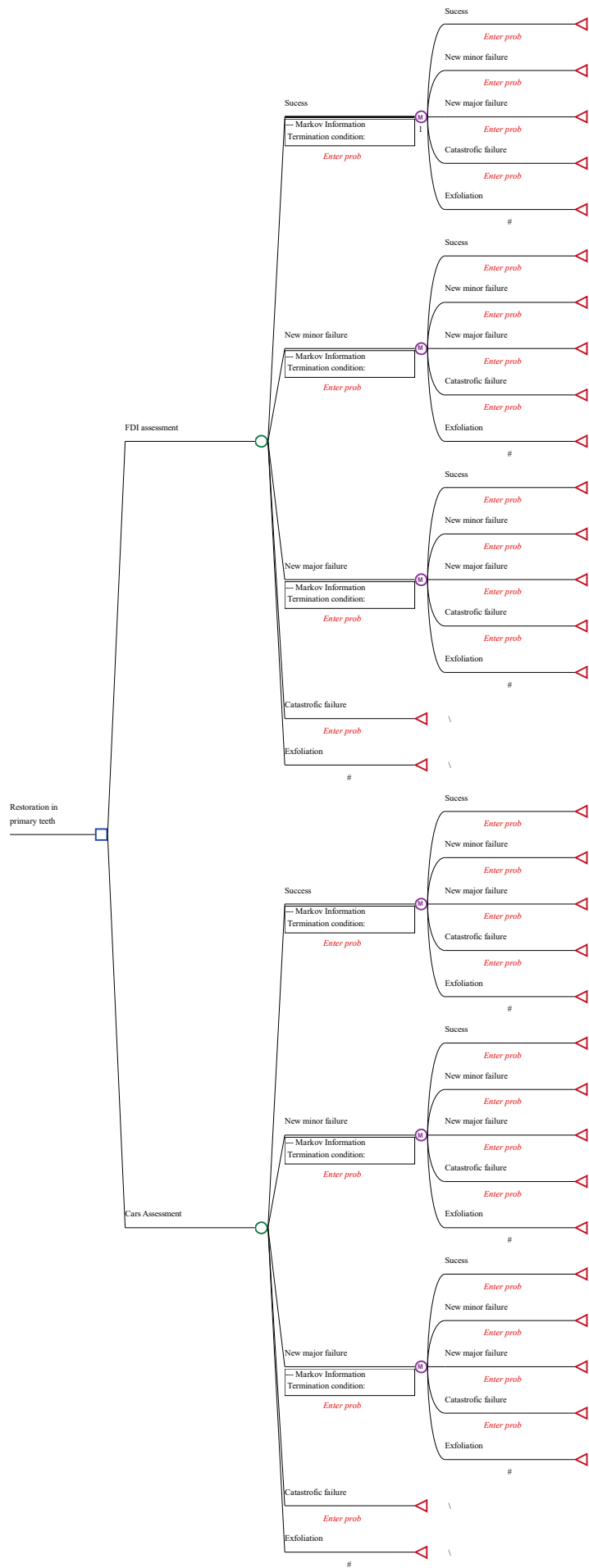

Supplement: Supplementary file 3 — Additional file 3:. Supplemental Material 3. First draft of a theoretical framework to construct an analytic Markov model for modelling strategies for primary tooth lifetime horizon [file 13063_2021_5722_MOESM3_ESM.pdf]

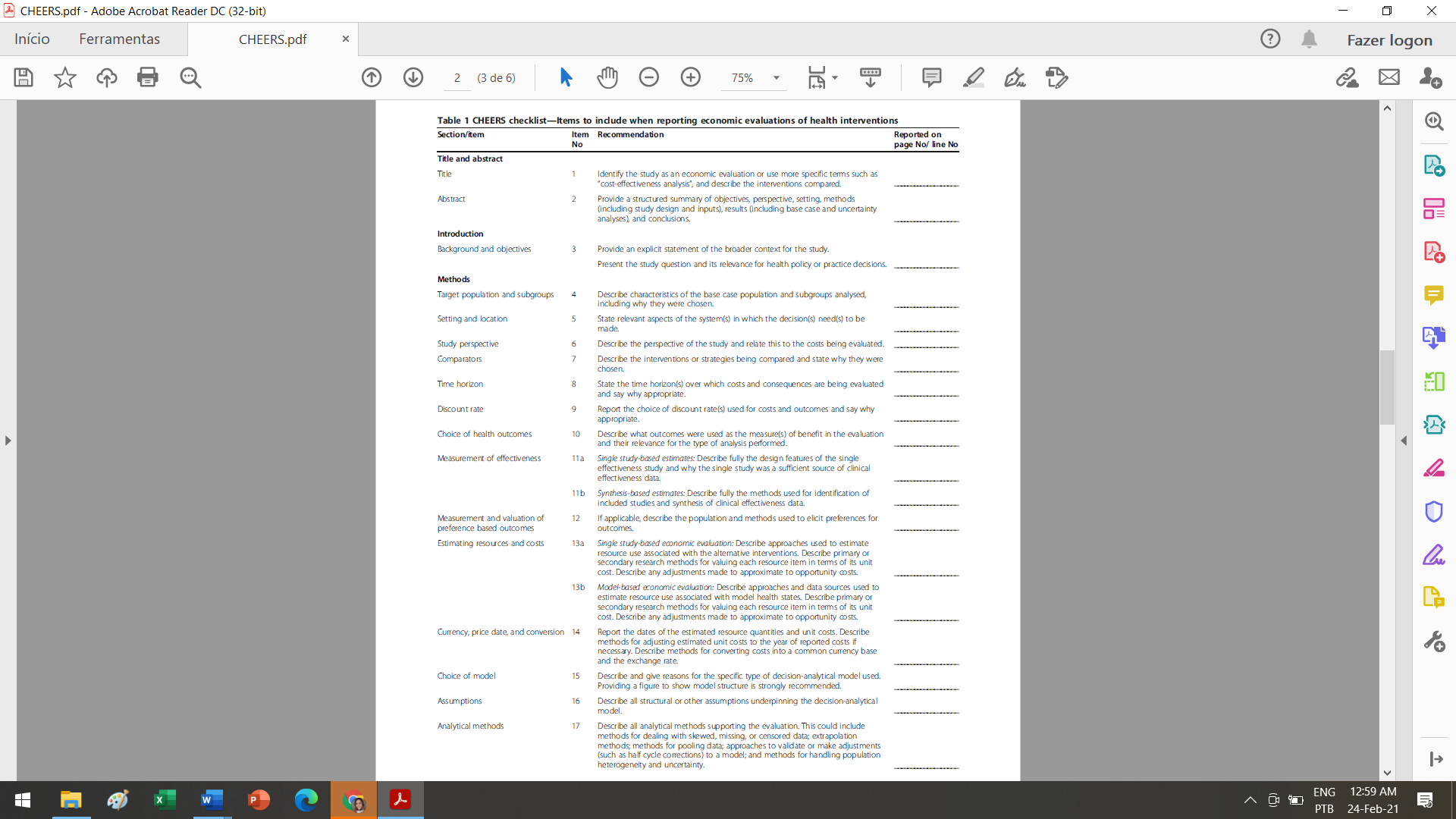


16-17

16-17

N/A

N/A

10-12

13-14

12

14

10-12

12

9

9

8

9

7

8

5

1

3


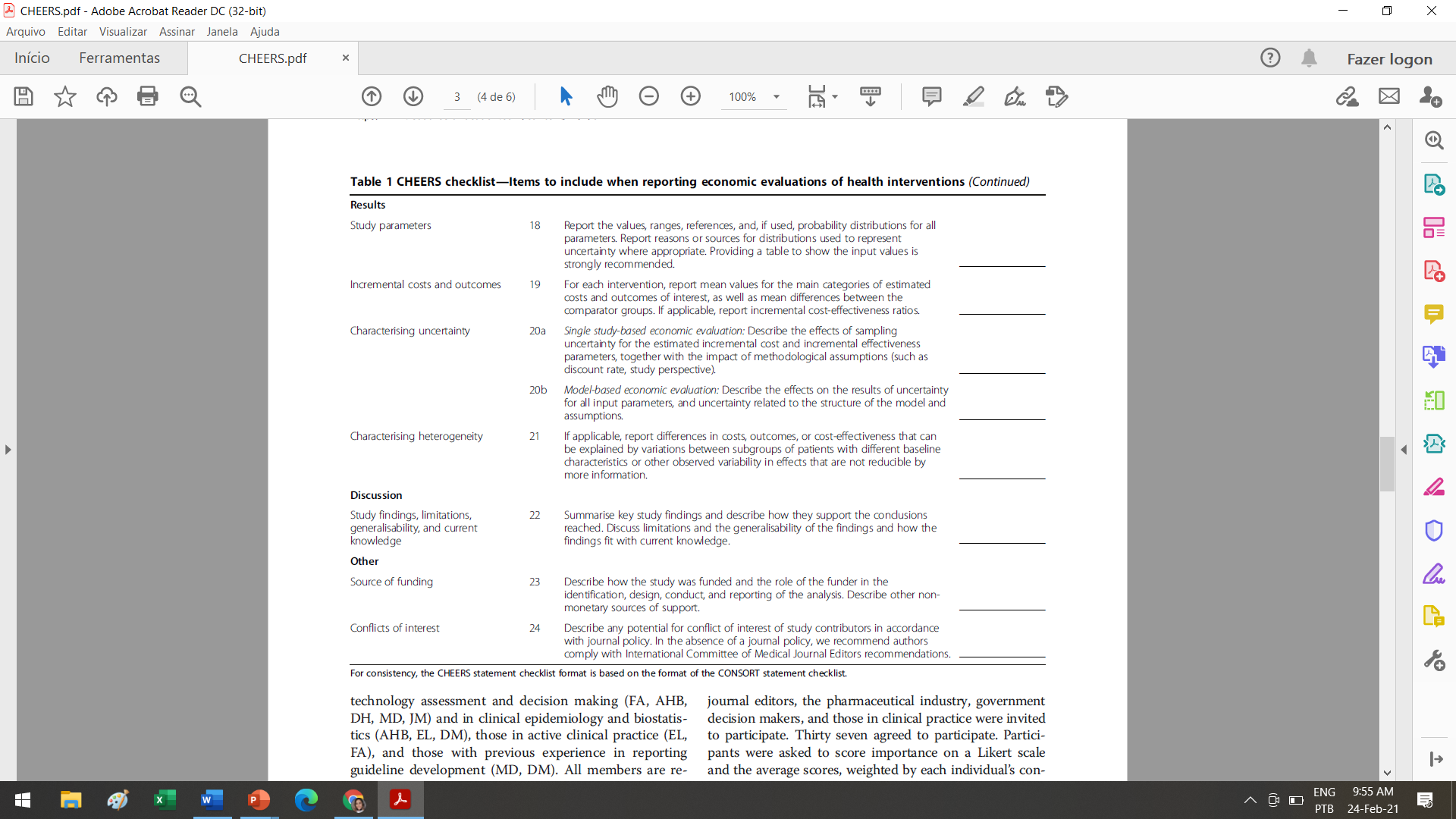


21

21

17-20

N/A

N/A

N/A

N/A

N/A

Supplement: Supplementary file 4 — Additional file 4:. EQUATOR network reporting checklist - Consolidated Health Economic Evaluation Reporting Standards (CHEERS) checklist. Note the items related to Results and Discussion (aspects related to findings) are not addressed since this is a health economic analysis plan. [file 13063_2021_5722_MOESM4_ESM.docx]
