# Supplementary material for: The economic impact of two diagnostic strategies in the management of restorations in primary teeth: a health economic analysis plan for a trial-based economic evaluation
Source: Trials. 2021 Nov 12;22:794. doi: 10.1186/s13063-021-05722-7 (PMC8586840; doi:10.1186/s13063-021-05722-7)
Supplement: Supplementary file 2 — Additional file 2:. Supplemental Material 2. Form used for resource measurement and subsequent cost estimation. [file 13063_2021_5722_MOESM2_ESM.docx]

| **Date** | **Procedure** | **Time** |
| --- | --- | --- |
|  |  |  |
|  |  |  |

| **X-RAY MATERIALS** | |
| --- | --- |
| Film hanger **(unit)** |  |
| Fixer **(50 ml)** |  |
| Periapical X-ray film **(unit)** |  |
| Child X-ray film **(unit)** |  |
| Film holders **(set)** |  |
| Developer **(50 ml)** |  |

| **SURGERY** | |
| --- | --- |
| Elevator **(unit)** |  |
| Blade **(unit)** |  |
| Scalpel Handle **(unit)** |  |
| Molt periosteal **(unit)** |  |
| 7 wax spatula **(unit)** |  |
| Suture **(unit)** |  |
| Forceps **(unit)** |  |
| Gauze **(1 swab)** |  |
| Carver Hollenbach **(unit)** |  |
| Needle holder **(unit)** |  |
| Saline water **(ml)** |  |
| Seringa descartável **(unit)** |  |
| Scissors **(unit)** |  |

| **ANESTHETICS & ISOLATION** | |
| --- | --- |
| Short needle **(unit)** |  |
| Ultra-short needle **(unit)** |  |
| Ainsworth rubber dam punch **(unit)** |  |
| Anesthethic cartridge **(unit)** |  |
| Topical anesthetic **(1 cm)** |  |
| Otsby dam frame **(unit)** |  |
| Dam clamp **(unit)** |  |
| Dental dam **(unit)** |  |
| Rubber dam fórceps **(unit)** |  |
| Cartridge syringe **(unit)** |  |

| **DISPOSABLES** | |
| --- | --- |
| 70% Alcohol **(50 ml)** |  |
| Bibs **(unit)** |  |
| Straw **(1/3 unit)** |  |
| Coffee cup **(unit)** |  |
| Wooden tongue depressor **(unit)** |  |
| Cling Film **(30 cm)** |  |
| Autoclave tape **(1 cm)** |  |
| Gloves **(1 pair)** |  |
| Mask **(unit)** |  |
| Protectice eyewear **(unit)** |  |
| Cotton Rolls **(unit)** |  |
| Syringe sleeve **(unit)** |  |
| Plastic clear gloves **(1 pair)** |  |
| Saliva ejector **(unit)** |  |
| Cap **(unit)** |  |

| **RESTORATIVES** | |
| --- | --- |
| Polyacrilic acid **(drop)** |  |
| Phosphoric acid **(1 cm)** |  |
| Single Bond Universal Adhesive **(drop)** |  |
| Round bur **(unit)** |  |
| Excavator **(unit)** |  |
| Wooden wedges **(unit)** |  |
| Spatula no. 1 **(unity)** |  |
| Carver Hollenbach **(unit)** |  |
| Matrix strip - 5mm **(1 cm)** |  |
| Matrix strip - 7 mm **(1 cm)** |  |
| Micro applicators **(unit)** |  |
| Dappen dish **(unit)** |  |
| Bulk Fill Restorative **(increment)** |  |
| Bulk Fill Flow **(increment)** |  |
| Z350 XT restorative **(increment)** |  |
| Riva Self Cure encaps. **(unit)** |  |
| Tira de poliéster **(1/2 unit)** |  |
| Petroleum jelly (**1cm)** |  |

| **PROPHY & EXAMINATION** |  |
| --- | --- |
| Mouth prop **(unit)** |  |
| Cheek retractor **(unit)** |  |
| Toothbrush **(unit)** |  |
| Prophy brush **(unit)** |  |
| Mirror **(unit)** |  |
| Disclosing dye - Replak **(drop)** |  |
| Floss **(10 cm)** |  |
| Examination kit (mouth mirror, probe, tweezers) |  |
| Prophy paste **(1 cm)** |  |
| Pumice powder **(1 portion)** |  |
| Dappen dish **(unit)** |  |
| Periodontal curettes **(unit)** |  |
| Prophy cup **(unit)** |  |

| **TOPICAL FLUORIDE APPLICATION** | |
| --- | --- |
| Duraphat **(1 cm)** |  |
| Wooden tongue depressor **(unit)** |  |
| Fluoride gel **(portion)** |  |
| Micro applicators **(unit)** |  |
| Fluoride tray **(unit)** |  |

| **FINISHING & POLISHING** | |
| --- | --- |
| Diamond bur **(unit)** |  |
| Polishing discs **(unit)** |  |
| Carbon film paper **(1/3 unit)** |  |
| Polishing strips **(unit)** |  |
| Abbrasive polishing strips **(1/2 unit)** |  |

**Dentist**, how was the child’s behavior during the procedure?

( ) A lot of difficulties

( ) Some difficulty

( ) Indifferent

( ) Cooperated reasonably

( ) Cooperated well

**WONG-BAKER FACIAL SCALE**

“How did you feel when treating your toothy?”.

**Dentista**, como a criança reagiu nesta sessão?

( ) Criou muita dificuldade

( ) Criou alguma dificuldade

( ) Indiferente

( ) Cooperou razoavelmente

( ) Cooperou bem

Tecido cariado do dente ___

**Restoration replacement:**

Decayed tissue underneath the old restoration:

None

Soft

Hard

**Decayed tissue**

Tooth: _______

None

Soft

Hard
